# Lymphocutaneous Nocardiosis in a Patient With Human Immunodeficiency/Tuberculosis Coinfection

**DOI:** 10.7759/cureus.22022

**Published:** 2022-02-08

**Authors:** Ranjan K Singh

**Affiliations:** 1 Internal Medicine, Anti-Retroviral Therapy Centre, District Hospital, Khagaria, IND

**Keywords:** branched filaments, lymphocutaneous abscess, nocardiosis, tuberculosis, hiv

## Abstract

*Nocardia* spp. are Gram-positive bacteria, which are acid-fast as well. Nocardiosis is characterized by abscess formation anywhere in the body, especially in the lungs, brain, and skin. The disease manifests as pulmonary disease, brain abscess, or disseminated lesions in immunocompromised individuals. However, skin involvement in the form of lymphocutaneous abscess is found in immunocompetent individuals. *Nocardia* spp. appear as thin, branched filaments in fine needle aspirate under the microscope. Diagnosis of the nocardiosis is done by fine needle aspiration cytology (FNAC) and identification through matrix-assisted laser desorption/ionization-time of flight mass spectrometry from aspirated materials. Our case is lymphocutaneous nocardiosis in a patient having human immunodeficiency (HIV)/tuberculosis coinfection.

## Introduction

*Nocardia *spp. are weak acid-fast and Gram-positive bacteria, which appear as thin branched filaments in fine-needle aspirate smears under the microscope. These bacterial infections are neglected especially in tropical countries and are of global concern. The annual incidence of Nocardial infection is 0.4 per 100,000 population at various centers and the incidence in HIV patients ranges between 3.4% and 16.7% [1]. Although the disease is easily missed, coinfections with tuberculosis in HIV patients are not uncommon [2]. The majority of primary cases manifest pulmonary disease; however, traumatically induced local cutaneous abscesses are not uncommon. Cutaneous presentations include cellulitis, lymphocutaneous abscess, or disseminated lesions with cutaneous infection.

The disease requires a high index of clinical suspicion to make a diagnosis. Diagnostic approaches include fine needle aspiration cytology (FNAC) and culture of aspirated abscess.

## Case presentation

**Figure 1:**
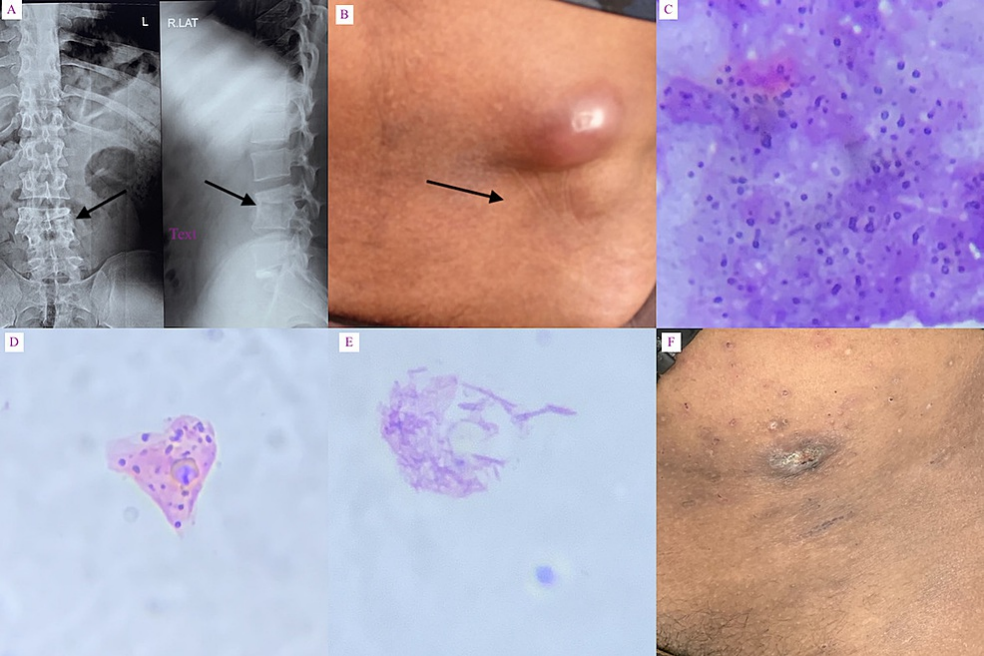
Panel A - collapse of intervertebral disc with wedging of L3/L4 vertebral bodies; Panel B - enlarged left inguinal lymph node and subcutaneous swellings over left lumbar region; Panels C-E - smear stained with Papanicolaou stain under the microscope (200x magnification) show granuloma formation, giant cell of foreign body and bunch of filaments appearing as cotton ball respectively; Panel F - healed lesion.

A 26-year-old male agriculture worker presented at our center with a history of multiple swellings over the left inguinal and left lumbar regions. The patient was on antiretroviral drugs including tenofovir, lamivudine, and dolutegravir for the past two years as he was seropositive for HIV-1. Seven months earlier, the patient experienced sciatic pain over the right lower limb. Chest X-ray revealed an obliterated right costophrenic angle. The spine X-ray (anterior-posterior, A-P/lateral views) (Figure 1A) showed wedging of the body of L3 vertebra and collapse of disc space between L3 and L4. Urine lipoarabinomannan testing and granulomatous lesions, partial collapse of L3 with discitis between L3/4 in MRI of spine favored tubercular lesion (cf. non-tubercular mycobacteria). The patient was started and continued on a four-drug anti-tuberculous regimen (isoniazid, INH; rifampin, RIF; ethambutol, EMB; and pyrazinamide, PZA) which alleviated pain in a few months. New distinct swellings were detected over the abdomen. 

Clinical examination revealed an enlarged left inguinal lymph node, which was soft and fluctuant. Two subcutaneous swellings, which were soft, fluctuant and non-tender, were detected in the left lumbar region (Figure 1B). The total leucocyte count was 12.4 x 109/L with neutrophil constituting 57% and hemoglobin levels at 11.8 g/dL. The HIV-1 load was 38 copies/mL and the CD4+ T cell count was 410/μL. We performed FNAC and prepared slides for microscopy. Aspirated material was sent for further laboratory culture studies. Smear under a microscope showed granuloma formation (Figure 1C), in addition to the multinucleated giant cell of foreign body type (Figure 1D) and a bunch of branched longitudinal filaments mimicking cotton ball (Figure 1E). Culture of pus with growth profile identification using matrix-assisted laser desorption/ionization-time of flight (MALTID-TOF) mass spectrometry showed *Nocardia nova*. He was treated with co-trimoxazole (25/5 mg/kg body weight) per day in divided doses. Swellings have regressed after one month of treatment (Figure 1F) and medication will be continued for another two months.

## Discussion

*Nocardia *spp. are opportunistic soil bacteria belonging to the Actinomycetes family. Among the 40 species identified, *N. nova*, *N. farcinica*, *N. cyriacigeorgica*, *N. brasiliensis*, and *N. abscessus* are routinely reported from clinical sources [3-4]. Nocardiosis is characterized by abscess formation anywhere in the body, especially in the lungs, brain, and skin. T cells provide a protective mechanism against the infection [5]. Invasive as well as disseminated infection occurs when immunity is compromised, for example, in HIV patients with a CD4+ T cell count < 250/μL [5]. A small number of cases with lymphocutaneous lesions have been found in immunocompetent individuals. 

In this case, *N. nova* caused lymphocutaneous lesions because antiretroviral medication brought the CD4 cell count close to normal level, making the patient somewhat immunosuppressed.

Among *Nocardia *spp., *N. brasiliensis* is the most common species isolated from a lymphocutaneous disease [2], while there are a few reports of *N. nova* causing the cutaneous disease [6-7].

Closely mimicking conditions include cold abscess, pyogenic abscess, eumycetoma, and sporotrichosis. In contrast to cutaneous abscesses caused by *Staphylococcous* or *Streptococcous* bacteria, cutaneous nocardiosis is indolent. The Nocardia infection, if spread to regional lymph nodes, causes a chain of nodular lesions called sporotrichoid nocardiosis resembling sporotrichosis. However, FNAC, culture, and profile analysis were used for differential diagnosis. *Nocardia* spp. can be identified in the fine-needle aspirate smear stained with Papanicolaou stain. Although granulomas are not well formed, multinucleated giant cells and branched filaments were detected under the microscope [8]. A number of conglomerate-like filamentous inclusions were detected as cotton balls [2]. *Nocardia *spp. grow in media for mycobacterial cultures. The species can be identified via molecular testing using techniques such as polymerase chain reaction, DNA probe, DNA sequencing, or matrix-assisted laser desorption ionization-time of flight (MALDI-TOF) mass spectrometry. MALDI-TOF mass spectrometry is used for rapid detection of *Nocardia *spp. and is comparable to molecular tests. 

## Conclusions

*Nocardia *spp. are soil-borne ubiquitous pathogens frequently infecting agricultural workers with defective cellular immunity. In contrast to invasive infections in patients with severe immune suppression, cutaneous infections are more common in immunocompetent or weakly immunosuppressed patients. Because this patient has a CD4 count of 410/μL and is somewhat immunosuppressed, lymphocutaneous lesion is expected in this group. Lymphocutaneous nocardiosis can be detected using FNAC and culture in agar media combined with MALDI-TOF mass spectrometry for identification of the species. *N. nova* is not an uncommon cause of primary cutaneous lesion and is sensitive to co-trimoxazole.
